# Incidence, Risk, and Clinical Course of New-Onset Diabetes in Long COVID: Protocol for a Systematic Review and Meta-Analysis of Cohort Studies

**DOI:** 10.2196/54853

**Published:** 2024-06-04

**Authors:** Ananya Sri Talanki, Neha Bajaj, Twinkle Trehan, Sathish Thirunavukkarasu

**Affiliations:** 1 College of Arts and Sciences Emory University Atlanta, GA United States; 2 Rollins School of Public Health Emory University Atlanta, GA United States; 3 Department of Family and Preventive Medicine Emory University School of Medicine Atlanta, GA United States; 4 Emory Global Diabetes Research Center Woodruff Health Sciences Center Emory University Atlanta, GA United States

**Keywords:** COVID-19, SARS-CoV-2, type 1 diabetes, type 2 diabetes, new-onset diabetes, long COVID, incidence, cohort studies, postacute sequela, systematic review, meta-analysis

## Abstract

**Background:**

COVID-19, an infectious disease pandemic, affected millions of people globally, resulting in high morbidity and mortality. Causing further concern, significant proportions of COVID-19 survivors endure the lingering health effects of SARS-CoV-2, the pathogen that causes COVID-19. One of the diseases manifesting as a postacute sequela of COVID-19 (also known as “long COVID”) is new-onset diabetes.

**Objective:**

The aim of this study is to examine the incidence of new-onset diabetes in patients with long COVID and assess the excess risk compared with individuals who tested negative for COVID-19. The study also aims to estimate the population-attributable fraction for COVID-19 as a risk factor for new-onset diabetes in long COVID and investigate the clinical course of new-onset diabetes cases.

**Methods:**

This is a protocol for a systematic review and meta-analysis. PubMed, MEDLINE, Embase, Scopus, and Web of Science databases will be systematically searched to identify articles published between December 2019 and July 2024. A comprehensive search strategy for each database will be developed using a combination of Medical Subject Headings terms, subject headings, and text words to identify eligible studies. Cohort studies and randomized controlled trials (only control arms) involving patients with COVID-19 of any age, with follow-up data on new-onset diabetes in long COVID, will be considered for inclusion. Controls will comprise individuals who tested negative for COVID-19, with or without other respiratory tract infections. Three independent reviewers (AST, NB, and TT) will perform article selection, data extraction, and quality assessment of the studies. A fourth reviewer (ST) will review the identified studies for final inclusion in the analysis. The random-effects DerSimonian-Laird models will be used to estimate the pooled incidence proportion (%), incidence rate of diabetes (per 1000 person-years), and risk ratio (with 95% CIs) for diabetes incidence.

**Results:**

A total of 1972 articles were identified through the initial search conducted in August 2023. After excluding duplicates, conducting title and abstract screening, and completing full-text reviews, 41 articles were found to be eligible for inclusion. The search will be updated in July 2024. Currently, data extraction is underway, and the meta-analysis is expected to be completed in August 2024. Publication of the study findings is anticipated by the end of 2024.

**Conclusions:**

The study findings should provide valuable insights to inform both clinical practice and public health policies regarding the effective management of new-onset diabetes in patients with long COVID.

**International Registered Report Identifier (IRRID):**

DERR1-10.2196/54853

## Introduction

### Overview

The COVID-19 caused by SARS-CoV-2 has had devastating effects on people’s health [1,2]. Globally, as of September 25, 2023, there have been more than 770 million confirmed cases of COVID-19, including nearly 7.7 million deaths [2].

While COVID-19 is primarily an infectious disease, its bilateral relationship with diabetes is of significant concern [3-5]. Diabetes, a global pandemic that has persisted for decades, is projected to affect 1.31 billion individuals worldwide by 2050 [6]. This chronic and debilitating condition affects multiple organs and systems, including the eyes, kidneys, nerves, heart, and blood vessels, consequently leading to increased morbidity and mortality [6]. Research has consistently shown that individuals with diabetes, whether preexisting or newly diagnosed, incur an increased risk of severe COVID-19, characterized by respiratory distress necessitating intensive care unit admission or mechanical ventilation, sepsis, or multi-organ failure [3,7,8]. Conversely, mounting evidence suggests that COVID-19 may precipitate the onset of diabetes during the acute phase of the illness [4,9,10] and the post-recovery phase [11-17], often referred to as “long COVID” [18].

Long COVID refers to a range of symptoms or conditions that persist or emerge 30 days or more after the initial infection or following hospital discharge (a proxy for clinical recovery) [18]. Research indicates that approximately 43% of individuals who recover from the acute phase of COVID-19 may develop long COVID [19]. Besides new-onset diabetes, patients with long COVID may experience other disorders such as myocarditis, arrhythmias, depression, anxiety, gastrointestinal disturbances, insomnia, muscle fatigue, and cognitive difficulties [19]. The development of diabetes in patients with long COVID can significantly affect their quality of life [20] and is likely to place additional strain on health care systems. Therefore, it is crucial to understand the extent of the risk of developing new-onset diabetes in patients with long COVID for early detection and effective management of this complication. Moreover, this understanding is essential for health care planning and resource allocation.

Previous systematic reviews examining the incidence and excess risk of new-onset diabetes in patients with long COVID (compared to individuals without COVID-19) are subject to several limitations. These limitations include the inclusion of new-onset diabetes cases that occurred during both the acute and post–COVID-19 phases [14], a small number of studies included in the meta-analysis [11,14,15], reliance solely on a single definition for COVID-19 diagnosis (such as the *International Classification of Diseases, Tenth Revision* [*ICD-10*] codes) [16], the absence of meta-analysis [13], and a focus limited to a specific type of diabetes [14]. Moreover, none of these studies have provided data on the clinical trajectory of new-onset diabetes cases or quantified the impact of COVID-19 as a risk factor for new-onset diabetes during the long COVID phase.

### Objectives

Our systematic review and meta-analysis objectives are (1) to examine the incidence of new-onset diabetes in patients with long COVID, (2) to evaluate the excess risk of new-onset diabetes in patients with long COVID compared with individuals who tested negative for COVID-19, (3) to estimate the population-attributable fraction (PAF) for COVID-19 as a risk factor for new-onset diabetes in long COVID, and (4) to investigate the clinical course of new-onset diabetes cases.

## Methods

### Overview

This protocol was developed following standard guidelines, including the Cochrane Handbook for Systematic Reviews [21] and the Preferred Reporting Items for Systematic Reviews and Meta-Analysis Protocols (PRISMA-P) [22]. The PRISMA-P checklist is provided in Multimedia Appendix 1. This study was registered in the International Prospective Register of Systematic Reviews (PROSPERO CRD42020200432) [23].

### Ethical Considerations

As this study does not have the direct involvement of human participants, an ethics committee approval is not required.

### Eligibility Criteria

#### Overview

This study followed the Population, Exposure, Comparator, Outcome, and Study Design (PECOS) framework [24] to formulate the eligibility criteria. All the criteria outlined apply to both the systematic review and meta-analysis, except for those pertaining to the clinical course of new-onset diabetes cases, which are not relevant to the meta-analysis.

#### Population

Patients of any age diagnosed with COVID-19, either clinically, using diagnostic codes (eg, *ICD-10* codes) [25], or by a positive SARS-CoV-2 test are included.

#### Exposure

Patients exposed to the SARS-CoV-2 virus infection are included.

#### Comparators

Comparators will consist of individuals who tested negative for SARS-CoV-2, either with or without other respiratory tract infections.

#### Outcomes

##### Overview

In the long COVID phase, as defined by the US Centers for Disease Prevention and Control (CDC) as 30 days or more after a COVID-19 diagnosis [18] or hospital discharge (a proxy for clinical recovery), the following outcomes will be assessed.

##### Primary Outcomes

The primary outcomes were, first, the incidence of new-onset diabetes, defined as the absence of previous diabetes history and meeting 1 of the following criteria: fasting plasma glucose (FPG) ≥126 mg/dL, 2-h post-load glucose (2-h PG, or postprandial glucose) ≥200 mg/dL, random blood glucose (RBG) ≥200 mg/dL, or hemoglobin A_1c_ (HbA_1c_) ≥6.5% [26,27], or diagnosed using *ICD-10* or similar codes for diabetes [25]. Second, the risk ratio for diabetes incidence in patients with COVID-19 compared with controls.

##### Secondary Outcomes

Secondary outcomes were PAF for COVID-19 as a risk factor for new-onset diabetes and clinical course of new-onset diabetes cases. To investigate the clinical course of new-onset diabetes cases during follow-ups in the post-COVID-19 phase, we will assess (1) persistence and regression of diabetes: the proportion of patients who remained diabetic (“persistence of diabetes”) and those who regressed to prediabetes or normoglycemia. Prediabetes is defined as FPG 100 or 110 to 125 mg/dL, 2-h PG 140 to 199 mg/dL, RBG 140 to 199 mg/dL, HbA_1c_ 5.7% or 6.0% to 6.4% [26,27], or diagnosed using *ICD-10* codes or similar codes [25]. Normoglycemia is defined as FPG <100 or 110 mg/dL, 2-h PG <140 mg/dL, RBG <140 mg/dL, HbA_1c_ <5.7% or 6.0% [26,27], or diagnosed using corresponding *ICD-10* codes or similar codes [25]); (2) antidiabetes medication use: the proportion of patients newly initiated on antidiabetes medications (oral drugs or insulin), those who continued, and those who discontinued such medications; (3) diabetes-related complications: the proportion of patients who developed diabetes-related complications such as retinopathy, neuropathy, nephropathy, or foot ulcers; (4) hospitalizations: the proportion of patients hospitalized due to diabetes-related reasons; (5) mortality: the proportion of patients who died due to poor glycemic control or diabetes-related complications; and (6) glycemic control: changes in mean glucose and HbA_1c_ levels over time.

### Study Design

Cohort studies and randomized controlled trials (only control arms) with follow-up data on new-onset diabetes in patients with long COVID will be considered. Cross-sectional and case-control studies, case reports, case series, letters, editorials, opinion articles, and comments will be excluded.

### Data Sources, Search Terms, and Search Strategy

PubMed, MEDLINE, Embase, Scopus, and Web of Science databases will be systematically searched to identify articles published from December 2019 to July 2024. Using the PECOS framework, a comprehensive search strategy for each database will be developed using a combination of Medical Subject Headings (MeSH) terms, subject headings, and text words to identify eligible studies. In PubMed, the MeSH terms will include “COVID-19,” “SARS-CoV-2,” “diabetes mellitus,” “cohort studies,” “longitudinal studies,” “follow-up studies,” “prospective studies,” “retrospective studies,” and “randomized controlled trial.” Additionally, the reference lists of selected studies, relevant narrative and systematic reviews, and the World Health Organization (WHO) COVID-19 research database [28] will be searched for additional relevant studies. No language restrictions will be applied. If articles written in languages other than English are identified, they will undergo translation by qualified translators. The search strategy utilized for the PubMed database is given in Multimedia Appendix 2.

### Study Selection

Three independent reviewers (AST, NB, and TT) will screen the titles and abstracts of studies retrieved from the database search. Afterward, they will screen the full text of the selected studies using the a priori inclusion and exclusion criteria to identify eligible studies. A fourth reviewer (ST) will review the identified studies for final inclusion in the analysis. The percent agreement for study selection between the three independent reviewers will be presented.

### Data Extraction

Data will be summarized as means (SDs) or medians (IQRs) for continuous variables and as frequencies (%) for categorical variables. We will design a data extraction form as per the Cochrane Handbook for Systematic Reviews [21]. Data on first author’s name, country, data sources, study design, study setting (community or hospital), number of participants with COVID-19, number of participants without COVID-19 or with other respiratory tract infections, age, sex, follow-up time, post–COVID-19 period, number of new cases of diabetes, diagnostic criteria for COVID-19, diagnostic criteria for diabetes, reported risk estimates, type of diabetes, covariates adjusted in regression models, and level of COVID-19 severity will be extracted by 3 independent reviewers (AST, NB, and TT). The reviewers will also collect data on the clinical course of new-onset diabetes cases, as listed under the eligibility criteria section. This includes data on the persistence of diabetes, regression to prediabetes or normoglycemia, mean glucose and HbA_1c_ levels, medication usage, development of diabetes-related complications, and hospitalizations and deaths due to diabetes-related reasons in the long COVID phase. Disagreements in the data extraction between the reviewers will be resolved by discussion or by a fourth reviewer (ST).

### Data Management

Articles retrieved from databases and other sources will be imported into the EndNote software (Clarivate), and a separate EndNote file will be created for each data source. These EndNote files will then be exported as “XML” files, which will be imported into the web-based Covidence (Veritas Health Innovation) platform [29]. The Covidence software will be used to remove duplicate records, screen articles, resolve disagreements between the reviewers, conduct data extraction using a customized template, perform risk of bias assessment, create a PRISMA (Preferred Reporting Items for Systematic Reviews and Meta-Analysis) flowchart, and export the eligible studies for conducting meta-analysis in RevMan Web (Cochrane) [30] and Stata software (version 18.0; StataCorp) [31].

### Risk of Bias and Certainty of Evidence

Three independent reviewers (AST, NB, and TT) will assess the risk of bias in the included studies using the Newcastle-Ottawa Scale (NOS) [32], and the Grading of Recommendations, Assessment, Development and Evaluation (GRADE) [33] framework to determine the certainty of the evidence. Consensus between the reviewers will be achieved by discussion or by a fourth reviewer (ST).

### Data Synthesis

The variances of incidence proportions (%) will be stabilized using the Freeman-Turkey Double Arcsine Transformation method [34]. The random-effects DerSimonian-Laird models [16,35] will then be used to pool the incidence proportion (%) and the incidence rate (per 1000 person-years) of diabetes across studies [35]. Meta-analysis to estimate the risk of developing new-onset diabetes among patients with long COVID compared with controls will use a 2-stage approach [21]. In the first stage, the aggregate effect estimate in each study will be extracted or derived. Subsequently, in the second stage, these estimates will be pooled using DerSimonian-Laird random-effects models [35] to compute pooled hazard ratios and their corresponding 95% CIs for diabetes incidence.

PAF in each study will be estimated using the below formula [36], and the PAFs across studies will be pooled using the random-effects models [35].




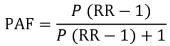




Where *P* stands for the proportion of patients with COVID-19 in the entire study population, RR stands for the risk ratio, and PAF is the proportion of all cases of a specific disease in a population attributable to a specific exposure [36]. The degree of between-study heterogeneity will be assessed using the Cochran *Q* test (*P*<.01 for heterogeneity) and Higgins *I*^2^ statistic (low: <25%, moderate: 25%-50%, and high: >50%) [37]. A meta-regression analysis will be performed to identify the sources of heterogeneity. Based on the data availability, subgroup analyses by age, sex, disease severity, hospitalization, follow-up time, the pandemic phase, time since the diagnosis of diabetes, type of diabetes, comorbidities, glycemic status (normal glucose tolerance or prediabetes), controls (no COVID-19 or those with other respiratory tract infections), race or ethnicity, and country will be performed. Publication bias will be assessed by funnel plots [38] and Egger test [39] if 10 or more studies are included in the meta-analyses. A sensitivity analysis will be conducted by excluding low-quality studies to assess the robustness of the primary findings, given the potential variability in study quality and risk of bias. A 2-sided *P*<.05 will be considered statistically significant. Analyses will be performed using RevMan Web (Cochrane) [30] and Stata (version 18.0; StataCorp) [31].

## Results

**Figure 1:**
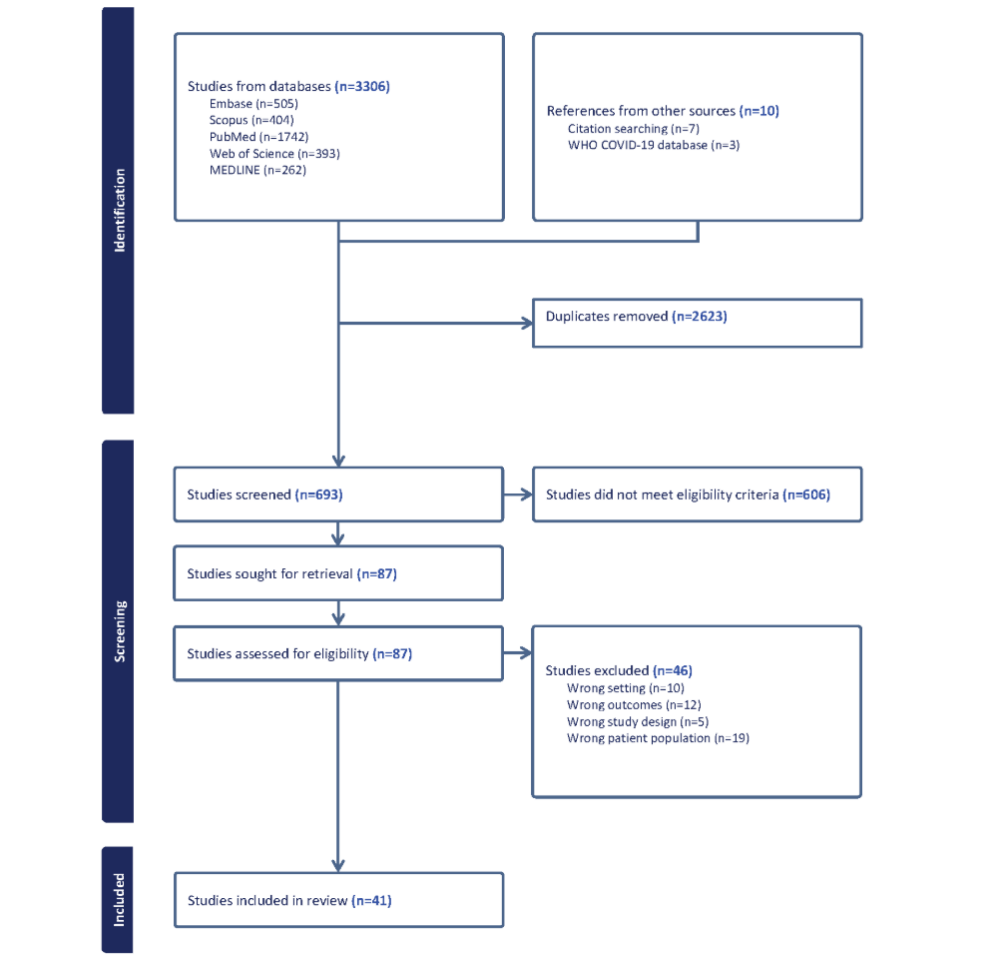
PRISMA flowchart. PRISMA: Preferred Reporting Items for Systematic Reviews and Meta-Analysis.

Figure 1 displays the PRISMA flowchart. The initial search was conducted in August 2023. A total of 3306 articles were identified through the database search, with an additional 10 articles from citation searching and the WHO COVID-19 database. After removing 1279 duplicates, the titles, and abstracts of 693 articles were screened. Among these, 606 articles were excluded as they did not meet the eligibility criteria. Subsequently, 87 articles underwent full-text reviews, with 46 articles excluded for various reasons, resulting in 41 eligible articles. Considering the dynamic nature of ongoing research on long COVID, we will be updating the search in July 2024 to retrieve articles that may have been published between August 2023 and July 2024 Table 1 shows the study timeline. Data extraction is currently underway, and the meta-analysis is expected to be completed in August 2024. Publication of the study findings is expected by the end of 2024.

**Table 1:** Study timeline.

Tasks	2023									2024							
	May	June	July	Aug	Sep	Oct	Nov	Dec	Jan		Feb	Mar	April	May	June	July to Sep	Oct to Dec
Formulation of study objectives	✓																
Development of search terms and strategies		✓	✓														
Database search				✓													
Title and abstract screening					✓	✓											
Full-text screening							✓	✓									
Data extraction									✓		✓	✓	✓				
Risk of bias and certainty of evidence assessment									✓		✓	✓	✓				
Database search update													✓			✓	
Meta-analysis															✓	✓	
Paper writing																✓	
Submission of paper for publication																	✓

## Discussion

### Principal Findings

Our systematic review and meta-analysis aim to examine the incidence and excess risk of new-onset diabetes in patients with long COVID compared with controls. The study will also estimate the PAF for COVID-19 as a risk factor for new-onset diabetes and investigate the clinical course of new-onset diabetes cases.

### Comparison With Previous Studies

Previous systematic reviews [11-16] have provided data on the burden of new-onset diabetes among individuals experiencing long COVID. However, each of these previous studies was constrained by certain limitations. The study conducted by Zhang et al [16] exclusively included studies where new-onset diabetes was diagnosed solely based on *ICD-10* codes. This methodology may have led to an underestimation of occurrences by overlooking cases identified through blood tests, potentially limiting the study’s accuracy in assessing the true burden of new-onset diabetes in patients with long COVID. In the meta-analysis by Bellia et al [12], a deviation from the widely accepted definition of long COVID was observed. By defining long COVID as 60 or more days from the diagnosis, instead of the recommended threshold of 30 or more days postdiagnosis [18], the study excluded potential diabetes cases occurring between 30 and 60 days postdiagnosis. Furthermore, Bellia et al’s [12] focus on the prevalence, rather than the incidence, of new-onset diabetes further narrows the scope of the study and limits its applicability in understanding the full extent of diabetes burden in patients with long COVID. The study conducted by Rahmati et al [14] is notable for its inclusion of both acute and patients with long COVID, potentially obscuring the unique risks and outcomes associated with the prolonged phase of COVID-19. The study by Banerjee et al [11] comprised a small number of studies (n=7), potentially constraining the statistical power of the subgroup analyses. Finally, the systematic review by Harding et al [13] lacked a meta-analysis component. While systematic reviews without meta-analyses still provide valuable insights by summarizing and synthesizing existing evidence, the absence of a quantitative synthesis may limit the study’s ability to quantify the overall effect size. In summary, these studies have contributed valuable insights into the burden of new-onset diabetes in the context of long COVID. However, their limitations underscore the need for further research efforts to address these gaps and provide a more comprehensive understanding of the impact of long COVID on diabetes incidence and outcomes.

### Potential Implications

Our study findings are expected to provide valuable insights for the effective management of patients with long COVID and new-onset diabetes. This includes the development of targeted interventions and therapies aimed at enhancing patient outcomes, along with tailoring interventions to subgroups at higher risk. Additionally, the insights garnered from our study have the potential to inform public health strategies, including vaccination campaigns, preventive measures, and postpandemic planning. Furthermore, our research is anticipated to contribute to the more effective allocation of health care resources.

### Strengths and Limitations

Our study will represent the largest investigation conducted to date in estimating the burden of new-onset diabetes in patients with long COVID. Furthermore, it will be the first to derive pooled estimates of the PAF for COVID-19 as a risk factor for new-onset diabetes in long COVID, while also examining the clinical trajectory of new-onset diabetes cases among patients with long COVID. Finally, standard statistical analysis will be used for conducting the meta-analysis per the Cochrane Handbook of Systematic Reviews [21]. However, our study also has some limitations. Heterogeneity among the included studies is expected due to potential variations in methodology, study settings, participant characteristics, diagnostic criteria for COVID-19 and new-onset diabetes, and the severity of COVID-19 cases. To address this, we will use random-effects meta-analysis and perform meta-regression and subgroup analyses to identify sources of heterogeneity. Despite our efforts to provide a comprehensive review, the dynamic nature of ongoing research on the long-term effects of COVID-19 may lead to additional studies emerging post-publication of our review. In conclusion, our research holds significant potential to provide valuable insights that can guide both clinical decision-making and public health policies for the effective management of new-onset diabetes in patients with long COVID.

## Appendix

Multimedia Appendix 1 PRISMA-P (Preferred Reporting Items for Systematic Reviews and Meta-Analysis Protocols) checklist.

Multimedia Appendix 2 Search strategy employed in PubMed.

## Abbreviations

2-h PG: 2-h post load glucose
CDC: Centers for Disease Prevention and Control
FPG: fasting plasma glucose
GRADE: Grading of Recommendations, Assessment, Development and Evaluation
HbA_1c_: hemoglobin A_1c_
*ICD-10*: *International Classification of Diseases, Tenth Revision*
NOS: Newcastle-Ottawa Scale
PAF: population-attributable fraction
PECOS: Population, Exposure, Comparator, Outcome, and Study Design
PRISMA: Preferred Reporting Items for Systematic Reviews and Meta-Analysis
PRISMA-P: Preferred Reporting Items for Systematic Reviews and Meta-Analysis Protocols
PROSPERO: International Prospective Register of Systematic Reviews
RBG: random blood glucose
WHO: World Health Organization

## Data Availability

All data generated or analyzed in this study are included in the published paper and Multimedia Appendices 1 and 2.
