# Supplementary material for: Incidence, Risk, and Clinical Course of New-Onset Diabetes in Long COVID: Protocol for a Systematic Review and Meta-Analysis of Cohort Studies
Source: JMIR Res Protoc. 2024 Jun 4;13:e54853. doi: 10.2196/54853 (PMC11185908; doi:10.2196/54853)
Supplement: Multimedia Appendix 2 [file resprot_v13i1e54853_app2.docx]

|  | Search terms | No. of articles |
| --- | --- | --- |
| 4 | **((((COVID-19[MeSH Terms]) OR (SARS-CoV-2[MeSH Terms])) OR ((((("COVID-19") OR ("SARS-CoV-2")) OR ("Coronavirus disease 2019")) OR ("Severe acute respiratory syndrome coronavirus 2")) OR (Severe acute respiratory syndrome coronavirus-2))) AND (("diabetes mellitus") OR (diabetes mellitus[MeSH Terms]))) AND (((((((((((("cohort studies") OR ("longitudinal studies")) OR ("follow-up studies")) OR ("prospective studies")) OR ("retrospective studies")) OR ("randomized controlled trials")) OR (cohort studies[MeSH Terms])) OR (longitudinal studies[MeSH Terms])) OR (follow-up studies[MeSH Terms])) OR (prospective studies[MeSH Terms])) OR (retrospective studies[MeSH Terms])) OR (randomized controlled trial[MeSH Terms]))** | 1,742 |
| 3 | **((((((((((("cohort studies") OR ("longitudinal studies")) OR ("follow-up studies")) OR ("prospective studies")) OR ("retrospective studies")) OR ("randomized controlled trials")) OR (cohort studies[MeSH Terms])) OR (longitudinal studies[MeSH Terms])) OR (follow-up studies[MeSH Terms])) OR (prospective studies[MeSH Terms])) OR (retrospective studies[MeSH Terms])) OR (randomized controlled trial[MeSH Terms])** | 2,881,593 |
| 2 | **("diabetes mellitus") OR (diabetes mellitus[MeSH Terms])** | 622,827 |
| 1 | **((COVID-19[MeSH Terms]) OR (SARS-CoV-2[MeSH Terms])) OR ((((("COVID-19") OR ("SARS-CoV-2")) OR ("Coronavirus disease 2019")) OR ("Severe acute respiratory syndrome coronavirus 2")) OR (Severe acute respiratory syndrome coronavirus-2))** | 413,077 |
